# Surface conditioning with *Escherichia coli* cell wall components can reduce biofilm formation by decreasing initial adhesion

**DOI:** 10.3934/microbiol.2017.3.613

**Published:** 2017-07-18

**Authors:** Luciana C. Gomes, Joana M. R. Moreira, José D. P. Araújo, Filipe J. Mergulhão

**Affiliations:** 1 LEPABE-Department of Chemical Engineering, Faculty of Engineering, University of Porto, Porto, Portugal; 2 CEFT-Department of Chemical Engineering, Faculty of Engineering, University of Porto, Porto, Portugal

**Keywords:** surface conditioning, *Escherichia coli*, mannose, myristic acid, palmitic acid, biofilm, parallel plate flow chamber, microtiter plate, computational fluid dynamics

## Abstract

Bacterial adhesion and biofilm formation on food processing surfaces pose major risks to human health. Non-efficient cleaning of equipment surfaces and piping can act as a conditioning layer that affects the development of a new biofilm post-disinfection. We have previously shown that surface conditioning with cell extracts could reduce biofilm formation. In the present work, we hypothesized that *E. coli* cell wall components could be implicated in this phenomena and therefore mannose, myristic acid and palmitic acid were tested as conditioning agents. To evaluate the effect of surface conditioning and flow topology on biofilm formation, assays were performed in agitated 96-well microtiter plates and in a parallel plate flow chamber (PPFC), both operated at the same average wall shear stress (0.07 Pa) as determined by computational fluid dynamics (CFD). It was observed that when the 96-well microtiter plate and the PPFC were used to form biofilms at the same shear stress, similar results were obtained. This shows that the referred hydrodynamic feature may be a good scale-up parameter from high-throughput platforms to larger scale flow cell systems as the PPFC used in this study. Mannose did not have any effect on *E. coli* biofilm formation, but myristic and palmitic acid inhibited biofilm development by decreasing cell adhesion (in about 50%). These results support the idea that in food processing equipment where biofilm formation is not critical below a certain threshold, bacterial lysis and adsorption of cell components to the surface may reduce biofilm buildup and extend the operational time.

## 1. Introduction

*Escherichia coli* is an important bacterial pathogen commonly implicated in outbreaks of foodborne diseases since it is capable of adhering to and form biofilms on food processing surfaces [1], leading to persistence and resistance to disinfection treatments [2]. The first step in the biofilm formation process is surface conditioning by molecules originating from the surrounding liquid such as ingredients from the culture medium [3] and components from cell lysis [4]. Following surface conditioning, free-floating bacterial cells will become attached, adhered and then retained on the surface [5]. It is at this point that bacteria start to form microcolonies and secrete extracellular polymeric substances (EPS) that are required for the interactions of the cells with the surface, with other cells and with other matrix components to develop the complex architecture of the biofilm. Bacteria can detach from the original biofilm and dispersed individual cells or clumps may spread into a new environment.

Removing biofilms in food processing plants is critical and it can be much more difficult than preventing their formation due to the high tolerance of sessile cells to cleaning agents [6],[7]. Moreover, the cleaning and disinfection methods currently used in industrial plants increase the process downtime and the production costs. Thus, an integrated strategy focused in the use of antibiofilm agents and other approaches to inhibit or disperse biofilms is being considered [8],[9],[10]. Although one might think that adsorbed cell components and related molecules can automatically enhance cell retention and reduce surface hygiene, it is known from several studies that proteins such as bovine serum albumin (BSA), gelatin, fibrinogen and pepsin adsorbed to surfaces may inhibit bacterial attachment [11],[12],[13]. Recently, it has been shown that surface preconditioning has potential to prevent bacterial adhesion to processing surfaces [14]–[18]. In order to elucidate about the process of bacterial contamination in dairy industry, Dat et al. [16] investigated the influence of surface conditioning with dairy by-products such as skimmed milk, buttermilk and butter serum (which possess different compositions) on the bacterial attachment behavior. It was found that stainless steel surfaces treated with these dairy by-products reduced cell adherence [15], which might be related to the milk composition, especially milk proteins [19]. Almost all acidic proteins were reported to reduce bacterial adherence, but basic and non-polar proteins enhanced it [20]. Additionally, the adherence-reducing ability of buttermilk and butter serum was proved to be better than skimmed milk due to the presence of substances associated with the milk fat globule membrane [15]. Other authors also revealed that the treatment of stainless steel surfaces with three types of milk decreased the adhesion of *Staphylococcus aureus* [16]. Studies using *E. coli* exopolysaccharides as surface coatings have provided further evidence that polysaccharides inhibit bacterial adhesion, possibly by modifying the physical properties of surfaces [21],[22]. Despite the knowledge on the effects of surface conditioning on bacterial adhesion, the impact of preconditioned surfaces on biofilm maturation is poorly understood.

Since the conditioned materials are usually integrated in engineered systems with particular hydrodynamic conditions, bacterial adhesion and the subsequent biofilm formation may also be affected by the fluid flow [23]. The hydrodynamics defines the rate at which macromolecules and cells are transported to the surface, the time they reside in the surface proximity, the oxygen and nutrient transport, and the mechanical shear stresses at the surface-fluid and fluid-biofilm interfaces [24],[25].

In a previous work, we have shown that different *E. coli* cell extracts (total cell extract, cytoplasm with cellular debris and periplasmic extract) inhibited biofilm formation under dynamic flow conditions [4]. The present study aims to evaluate the effect of surface conditioning with representative components of cell wall (mannose, myristic acid and palmitic acid) on *E. coli* biofilm formation. A screening of the most important conditioning agents affecting biofilm formation was performed in agitated 96-well microtiter plates in order to take advantage of the high throughput of this platform. Then, the effect of the most relevant concentrations was evaluated on biofilm formation and bacterial adhesion assays performed in a parallel plate flow chamber (PPFC) under the same shear stress obtained in the 96-well microtiter plate. The scalability of the results produced in this small scale system (with a different flow topology from traditional flow systems) and the possibility of its application to study the biofilms developed in industrial settings are discussed.

## 2. Materials and Methods

### 2.1. Numerical simulations

**Figure 1.**
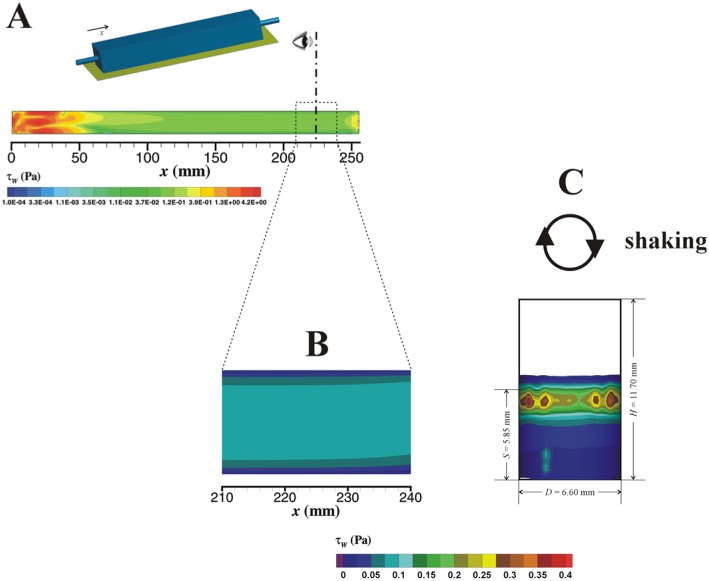
Time averaged wall shear stress (*τ_w_*) in a PPFC (A and B) and in a well of a 96-well microtiter plate (C). A flow rate of 11 ml s^−1^ was used for the simulation in the PPFC. (A) Wall shear stress on the bottom surface of the PPFC. (B) Detail of the wall shear stress in the visualization zone. (C) Wall shear stress in a well of a 96-well microtiter plate placed in an orbital shaker with 50 mm diameter at 150 rpm; the well dimensions (*D* and *H* and the liquid level at stationary condition (*S*) are indicated.

Numerical simulations were made in Ansys FLUENT CFD package (version 14.5; Ansys, Inc., USA) for two distinct cases: a cylindrical well of a 96-well microtiter plate (diameter (*D*) of 6.6 mm and height (*H*) of 11.7 mm, maximum volume of 0.36 ml, Figure 1C) subjected to an orbital motion with amplitude of 50 mm and shaking frequency of 150 rpm [26]; a PPFC unit (with a cross section of 8 × 16 mm and a length of 254 mm) at different flow rates. The three-dimensional geometries of the domains were built in Design Modeller 14.5 (Ansys, Inc., USA) and discretized by Meshing 14.5 (Ansys, Inc., USA) into grids of 18,876 hexahedral cells (in the case of microwell) and 1,694,960 hexahedral cells (in the case of PPFC).

In the simulation of the well, we were dealing with a two-phase flow scenario, so the volume of fluid (VOF) methodology was used to track the liquid-gas interface and the precise location of the interface was obtained by the Geo-Reconstruct method. The surface tension effects were modelled by the continuum surface force, and an accelerating reference frame and the circular orbital motion were implemented. The simulation was initialized with the well filled with 200 µl of liquid and the remaining volume consisting of air. The properties of water and air at 30 °C were used for the liquid and gas phases, respectively. The no slip boundary condition and a contact angle of 83° were fixed for all walls. The velocity-pressure coupled equations were solved by the PISO algorithm, the QUICK scheme was used for the discretization of the momentum equations and the PRESTO! scheme was applied for pressure discretization. A physical time of 5 s was simulated with a fixed time step of 2.5 × 10^−4^ s.

In the case of the PPFC unit, several simulations were performed for the PPFC with the purpose of determining the liquid flow rate that yields an average wall shear stress (*τ^w^*) in the visualization zone similar to the one obtained inside the wells at the shaking conditions used in this work [27]. The flow rate conditions of these simulations led to flow under turbulent regime (Reynolds number higher than 3500), therefore the SSL k-ω model with low Reynolds corrections was applied. The whole set of PPFC simulations was performed in transient mode due to the unsteadiness associated with the jet flow that forms at the inlet and to assure convergence. An initial condition of zero velocity was set for the whole domain and the boundary conditions comprehended a uniform velocity profile at the inlet, a zero relative pressure at the outlet and a no slip condition for all the walls. The fluid was assumed to be water at 30 °C. Similarly to the simulation of the microwell, the solution methods used were PISO, QUICK and the PRESTO!. A physical time of 2 s was simulated and a fixed time step of 10^−4^ s was used.

### 2.2. Conditioning agents

Three components representative of the *E. coli* cell wall were tested as conditioning agents: mannose, myristic acid and palmitic acid. Mannose is a sugar monomer which is typically found on the walls of bacterial cells [28]. Furthermore, it is one of the predominant monosaccharide components detected on the cell surfaces of various enteropathogenic *E. coli* serotypes [29]. The two saturated fatty acids palmitic acid (C16:0) and myristic acid (C14:0) are the dominant ones in the *E. coli* cell wall [30],[31], consisting of more than 50% of the fatty acid content in continuous cultures [31].

D-(+)-mannose (Fluka Analytical, cat. no. 63580, USA) was prepared in sterile distilled water at concentrations of 0.5, 1, 5, 10 and 100 g l^−1^. Given the low solubility of the palmitic (Merck KGaA, cat. no. 800508, Germany) and myristic acid (Fluka Analytical, cat. no. 70082, USA) in water, concentrated solutions (25 g l^−1^) were prepared using absolute ethanol (PanReac AppliChem, Germany) from which working solutions of 2.5 × 10^−4^, 2.5 × 10^−3^, 0.025, 0.25 and 2.5 g l^−1^ (below the micellar concentration) were prepared in distilled water. The pH of myristic acid solutions was 6.58 ± 0.16, while the pH of palmitic acid solutions was 6.68 ± 0.12.

### 2.3. Bacteria and culture conditions

*E. coli* JM109 (DE3) from Promega (USA) was used in this study since it had already shown a good biofilm forming capacity in different platforms [32],[33],[34]. An overnight culture of this strain was prepared by adding 500 µl of a glycerol stock (kept at –80 °C) to 0.2 l of inoculation media (5.5 g l^−1^ glucose, 2.5 g l^−1^ peptone, 1.25 g l^−1^ yeast extract in phosphate buffer (1.88 g l^−1^ KH_2_PO_4_ and 2.60 g l^−1^ Na_2_HPO_4_, pH 7.0) and incubating at 30 °C with orbital agitation [33]. Cells were then harvested by centrifugation (3202 g, 10 min) and washed twice with citrate buffer 0.05 mol l^−1^, pH 5.0 [34]. The pellet was resuspended and diluted in the same buffer in order to reach an optical density (OD) of 0.1 at 610 nm (corresponding to a cell concentration of 7.6 × 10^7^ cell ml^−1^). This suspension was used for adhesion and biofilm formation assays in the PPFC and 96-well microtiter plates.

### 2.4. Microtiter plate assay

Six wells of sterile 96-well polystyrene, flat-bottomed microtiter plates (Orange Scientific, cat. no. 4430100N, USA) were filled with 200 µl of the conditioning agent at each desired concentration. The plates were incubated for 1 h at 30 °C with orbital agitation (50 mm of shaking diameter at 150 rpm). After surface conditioning, each well was washed [35],[36] with 200 µl of citrate buffer and filled with 200 µl of the cellular suspension previously prepared. Clean wells were also washed with citrate buffer and inoculated with the cellular suspension (reported as “control”). The plates were incubated at 30 °C with agitation (50 mm of shaking diameter at 150 rpm) for 24 h to promote biofilm formation. Biofilm amount was assessed by staining with crystal violet [26] and expressed as OD_570 nm_ values.

### 2.5. Parallel plate flow chamber assay

The conditioning agents which have shown some effect on biofilm formation in the microtiter plate assay were chosen at the most effective concentration to be tested in the PPFC with the aim of assessing their effect on adhesion (after 30 min) and biofilm formation (after 24 h). Adhesion assays were not performed in the microtiter plate due to the detection limit of the crystal violet method.

To conduct the assays, the PPFC was connected to a tank and a centrifugal pump by a tubing system [37]. The PPFC has recesses in its bottom for the introduction of round polystyrene coupons (1 cm diameter) so they become flush with the surface. Before being introduced into the PPFC, the coupons were soaked for 5 min in a commercial detergent (Sonasol Pril; Henkel Ibérica, S.A., Spain), then immersed in a sodium hypochlorite solution (3%, v/v) for more 5 min and aseptically rinsed in distilled water for 20 min. The PPFC was conditioned for 1 h at a flow rate of 11 ml s^−1^, which corresponds to the average *τ^w^* operated in the microtiter plates (0.07 Pa). Then the flow cell was washed with citrate buffer and filled with the *E. coli* suspension (OD_610 nm_ = 0.1) that circulated through the PPFC at a flow rate of 11 ml s^−1^. Unconditioned polystyrene coupons were used as control. After 30 min of bacterial adhesion and 24 h of biofilm formation, the coupons were retrieved from the PPFC and total bacterial counts were obtained by direct staining with 4′,6-diamidino-2-phenylindole (DAPI; Sigma-Aldrich, Portugal) [38]. Cells were visualized under an epifluorescence microscope (Eclipse LV100; Nikon, Japan) equipped with a filter block sensitive to DAPI fluorescence (359-nm excitation filter in combination with a 461-nm emission filter). A minimum of 10 fields from each coupon were counted and used to estimate the number of cells per cm^2^ of coupon area. Since the biofilms formed were mainly composed of cells, the results obtained by the DAPI staining method are directly comparable to those obtained by the crystal violet assay.

### 2.6. Surface hydrophobicity

The hydrophobicity of bare polystyrene and polystyrene conditioned with myristic and palmitic acid was evaluated considering the Lifshitz-van der Waals acid base approach [39]. The contact angles were determined automatically by the sessile drop method in a contact angle meter (OCA 15 Plus; Dataphysics, Germany) using water, formamide and α-bromonaphtalene (Sigma-Aldrich Co., Portugal) as reference liquids [40]. The surface tension components of the reference liquids were taken from literature [40]. For each surface, measurements with each liquid were performed at 25 ± 2 °C. The model proposed by van Oss [39] indicates that the total surface energy (*γ^Tot^*) of a pure substance is the sum of the Lifshitz-van der Waals components of the surface free energy (*γ^LW^*) and Lewis acid-base components (*γ^AB^*):

$$\gamma^{Tot}=\gamma^{LW}+\gamma^{AB}$$ (1)

The polar AB component comprises the electron acceptor (*γ*^+^) and electron donor (*γ*^−^) parameters, and is given by:

$$\gamma^{AB}=2\sqrt{\gamma^{+}\gamma^{-}}$$ (2)

The surface energy components of a solid surface (*s*) are obtained by measuring the contact angles (*θ*) with the three different liquids (*l*) with known surface tension components, followed by the simultaneous resolution of three equations of the type:

$$(1+\cos\theta )\gamma_{l}{}^{Tot}=2\left(\sqrt{\gamma_{s}^{LW}\gamma_{l}^{LW}}+\sqrt{\gamma_{s}^{+}\gamma_{l}^{-}}+\sqrt{\gamma_{s}^{-}\gamma_{l}^{+}}\right)$$ (3)

The degree of hydrophobicity of a given surface is expressed as the free energy of interaction (Δ*G*) between two entities of that surface immersed in a polar liquid (such as water (*w*) as a model solvent). Δ*G* was calculated from the surface tension components of the interacting entities, using the equation:

$$\Delta G=-2{\left(\sqrt{\gamma_{s}^{LW}}-\sqrt{\gamma_{w}^{LW}}\right)}^{2}+4\left(\sqrt{\gamma_{s}^{+}\gamma_{w}^{-}}+\sqrt{\gamma_{s}^{-}\gamma_{w}^{+}}-\sqrt{\gamma_{s}^{+}\gamma_{s}^{-}}-\sqrt{\gamma_{w}^{+}\gamma_{w}^{-}}\right)$$ (4)

If the interaction between the two entities is stronger than the interaction of each entity with water, Δ*G* < 0 mJ m^−2^, the material is considered hydrophobic; if Δ*G* > 0 mJ m^−2^, the material is hydrophilic.

### 2.7. Statistical analysis

Bacterial adhesion and biofilm quantification results are averages from three independent experiments performed for each conditioning agent and concentration. Paired *t*-test analysis were performed based on a confidence level of 95% to determine whether or not there was a significant difference between the results and the control (differences reported as significant for *P* values < 0.05 and marked with ★).

## 3. Results

### 3.1. Numerical simulations

The wall shear stress (*τ_w_*) can strongly impact the biofilm formation [37], so the focus of the numerical data analysis was placed on the results obtained for this specific hydrodynamic feature. Simulation data of the *τ_w_* field along the PPFC and in a well of a 96-well microtiter plate is collected in Figure 1. In Figure 1C, a front view of the time averaged *τ_w_* distribution in the internal wall of the well (in an orbital shaker with 50 mm diameter at 150 rpm) is shown. The shear stress is unequally distributed throughout the wetted surface and higher values are found in the liquid side near the interface. Here there are spots with relative *τ_w_* maxima that are associated with the presence of unstable vortices near the wall. Based on the data presented in Figure 1C, an average *τ_w_* value of 0.07 Pa was calculated for the agitated microwell.

In the case of the PPFC, and for an inlet flow rate of 11 ml s^−1^, the entire ***τ_w_*** field in the bottom surface of the channel (width of 16 mm and length of 254 mm) was plotted (Figure 1A). Due to the jet flow originated at the inlet expansion, the higher values of *τ_w_* occur for *x* < 50 mm. For *x* around 120 mm, the wall shear stress seems to stabilize, and in the visualization zone the flow is in fully developed state and the corresponding hydrodynamic features are stable. The representation in Figure 1B was obtained by zooming panel A to the dimensions of the visualization zone and changing the color map to the one used in Figure 1C to facilitate comparison. In Figure 1B, it is observed that *τ_w_* is constant in central regions of the surface, however it decreases substantially as the lateral edges are approached due to the reduction of the velocity gradient in corner regions (junction of two perpendicular walls). The average *τ_w_* value calculated for the visualization zone of PPFC is 0.074 Pa, which is similar to the one determined in the simulation of the well. This indicates that these two different platforms induce a similar hydrodynamic influence on the biofilm, despite the volumetric scale-up of 100 fold.

### 3.2. Biofilm formation

**Figure 2.**
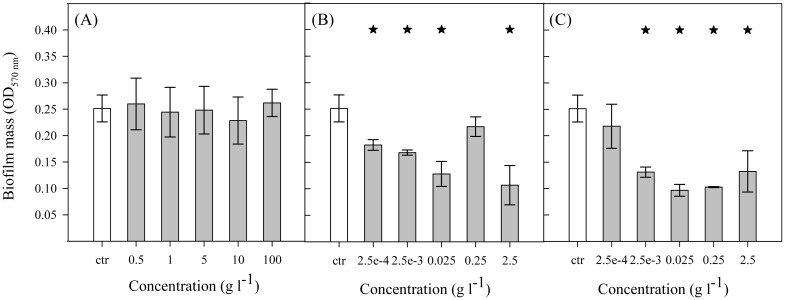
Biofilm formation (OD_570 nm_) in 96-well microtiter plates preconditioned with (A) mannose, (B) myristic acid and (C) palmitic acid at different concentrations (
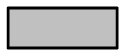
). Biofilm formed on unconditioned surface was used as control (ctr, 
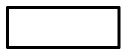
). The mean values ± standard deviation for three independent experiments are illustrated. Statistical analysis is represented with an asterisk (★) for a confidence level greater than 95% (*P* < 0.05).

**Figure 3.**
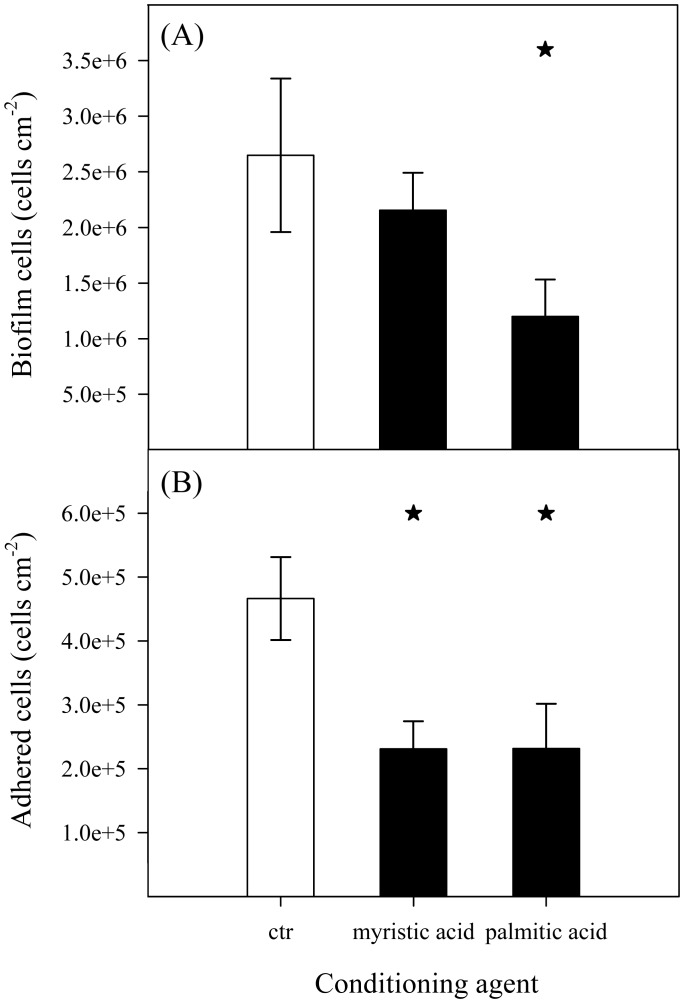
Number of adhered cells per cm^2^ in the PPFC after (A) 24 h and (B) 30 min on polystyrene surfaces preconditioned with myristic and palmitic acid at 0.025 g l^−1^ (
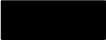
). Cells adhered on unconditioned surface were used as control (ctr, 
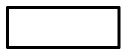
). The mean values ± standard deviation for three independent experiments are illustrated. Statistical analysis is represented with an asterisk (★) for a confidence level greater than 95% (*P* < 0.05).

A 96-well microtiter plate and a PPFC were used to investigate the effect of surface conditioning with cellular components on *E. coli* adhesion and biofilm formation. Microtiter plates were used for screening (Figure 2) in order to take advantage of the high-throughput of this platform. Since flow systems are typical of industrial settings, the most relevant conditions originated from the screening were then tested in the PPFC (Figure 3). The two platforms were operated in conditions that promoted a similar average shear stress in the wetted surface of a well and in the visualization zone of the PPFC (0.07 Pa).

Figure 2 presents the biofilm quantification results when mannose, myristic acid and palmitic acid were used as surface conditioning agents. While in the case of mannose (Figure 2A) the amount of biofilm formed in the conditioned wells was similar to the control for all tested concentrations (*P* > 0.05), for myristic and palmitic acid (Figure 2B and Figure 2C, respectively) a lower amount of biofilm was detected for most of the concentrations tested (*P* < 0.05 for 4 out of 5 cases). However, myristic and palmitic acid results do not show a concentration dependent behavior. Overall, concentrations of 0.025 g l^−1^ of these conditioning agents were the most effective, with a biofilm reduction of 49% for myristic acid and 62% for palmitic acid.

After the microplate assays, myristic acid and palmitic acid, which were the conditioning agents with the greatest impact on biofilm formation (Figure 2), were tested in the PPFC to study their effect on cell adhesion and biofilm formation (Figure 3). Surfaces were preconditioned with myristic and palmitic acid at the most effective concentration (0.025 g l^−1^). One of the goals of the PPFC assay was to verify if the results obtained in 96-well microtiter plates were scalable to a flow cell system. Furthermore, we aimed to understand if the biofilm reduction was due to a lower initial adhesion or to other events occurring during biofilm growth. Results showed that 24-h biofilm formation under flow (Figure 3A) was reduced by both myristic acid (in about 19%, although not statistically significant compared to control) and palmitic acid (in about 55%, *P* < 0.05). They agreed with the results obtained in the microtiter plate assays, being the palmitic acid more effective in inhibiting biofilm growth than the myristic acid. With regard to adhesion (Figure 3B), a decrease of about 50% was observed on surfaces preconditioned with both cellular components when compared to the unconditioned surface. It is also important to note that, for palmitic acid, similar reduction values were obtained for initial adhesion and biofilm formation (Figure 3).

The physicochemical characterization of the polystyrene surface before and after conditioning was made by contact angle measurement (Table 1). From the free energy of interaction (Δ*G*), it is possible to observe that the bare polystyrene and the polystyrene conditioned with myristic and palmitic acid at the concentration with the greatest impact on biofilm formation (conditions used in the PPFC assay) are hydrophobic surfaces (Δ*G* < 0 mJ m^−2^). However, the unconditioned surface is slightly more hydrophobic than the conditioned surfaces. Regarding the van der Waals forces apolar component (*γ^LW^*), it is possible to conclude that the three surfaces have a similar value. In what concerns to the polar surface components (*γ^+^* and *γ^−^*), results show that the bare polystyrene is a monopolar surface, being an electron donor, while both conditioned surfaces are polar surfaces, being simultaneously electron donors and acceptors.

**Table 1.** Contact angles with water (*θ_w_*), formamide (*θ_F_*) and α-bromonaphthalene (*θ_B_*), surface tension parameters (*γ^LW^, γ^+^* and *γ^−^*) and hydrophobicity (Δ*G*) of the bare polystyrene and conditioned surfaces. Values are means ± SDs of three independent experiments.

	Contact angle (°)			Surface tension propertiesmJ m^−2^)			Hydrophobicity (mJ m^−2^)

	*θ_w_*	*θ_F_*	*θ_B_*	*γ^LW^*	*γ^+^*	*γ^−^*	Δ*G*
Bare polystyrene	81.1 ± 0.682	64.3 ± 1.24	24.6 ± 1.11	40.5	0.000	7.95	–50.8
Polystyrene + myristic acid	74.5 ± 0.551	52.1 ± 1.27	29.3 ± 0.894	38.9	0.337	7.79	–45.3
Polystyrene + palmitic acid	72.2 ± 0.567	49.1 ± 0.918	31.0 ± 0.413	38.3	0.621	8.44	–41.2

## 4. Discussion

The first event that occurs when materials are placed in the food environment is the appearance of a so-called conditioning film, typically consisting of molecules from the surrounding medium and from cell lysis. The conditioning film can promote or inhibit the adhesion and proliferation of bacteria, depending on the process conditions. In this work, the influence of surface conditioning with components of cell wall on *E. coli* biofilm formation was assayed in two different platforms. The screening of conditioning agents was first conducted in agitated 96-well microtiter plates and two inhibiting components were identified. Then, the effect of the most relevant concentrations of these components was verified in a PPFC using the same adhesion material (polystyrene) and under the same average wall shear stress obtained in the microtiter plate (as determined by CFD).

Similar reduction values were obtained in both biofilm forming platforms, demonstrating that the average *τ_w_* is a suitable scale-up parameter from 96-well microtiter plates to larger scale flow cell systems as the PPFC used in the present study. Nevertheless, it is important to take into account that the flow topologies in the two platforms are very different and that this difference may affect biofilm formation [41] at high shear stresses. Our results show that when low shear stress conditions are considered, the average *τ_w_* captures the biofilm formation behavior that is obtained in two different biofilm reactors, being a good scale-up factor from high-throughput devices like 96-well microtiter plates, which are extensively used for biofilm studies [26],[42],[43],[44], to flow systems found in industry. We hypothesize that flow topology variations occur at a much larger scale than the dimensions of the bacterial cells, which renders them almost insensitive to these variations, particularly at low shear stress values.

In this work, mannose did not have any effect on bacterial biofilm formation. In contrast, Trautner et al. [45] revealed that modifying silicone surfaces to present mannose ligands for the type 1 fimbriae of *E. coli* promoted the formation of *E. coli* 83972 biofilms (4.4-fold more denser than on unmodified surfaces), thereby establishing a protective biofilm that reduced pathogenic *Enterococcus faecalis* colonization. Also, in previous work, Rodrigues and Elimelech [46] had already observed that type 1 fimbriae are critical on *E. coli* K12 biofilm development because these appendages are able to recognize the mannose-rich EPS synthesized by the bacterium, which acts as a “conditioning film” for the anchorage of fimbriae. However, they indicate that mannose was not important for *E. coli* adhesion to glass surfaces since type 1 fimbriae were not required for initial adhesion under the tested conditions [46]. These authors also found that the concentration of D-mannose influences biofilm density in microtiter plates. The optimum production of biofilm was achieved at 1% D-mannose concentration, but it was reduced at higher concentrations (5%) [46]. On the other hand, Pratt and Kolter [47] discovered that *E. coli* K12 attachment to polyvinyl chloride is hindered by the presence of mannose, although this compound did not inhibit biofilm growth. The results obtained in the present study and in other published works [45],[46],[47] suggest that the influence of mannose on *E. coli* adhesion and biofilm formation depends on the existing abiotic material. Although there are some reports about the interactions between *E. coli* and the mannose present on abiotic surfaces, the major part of the current knowledge was obtained from the *E. coli* colonization of host tissues [48]–[51]. Studies using *E. coli* K12 have shown that, from about a dozen sugars tested, only D-mannose and its derivatives inhibited (at low concentrations) the attachment of bacteria to human buccal epithelial cells [52] or displaced the pre-attached bacteria from the cells [50],[51]. The simplest explanation of the inhibitory action of mannose is that it serves as an analogue of fixed D-mannose-like residues on the surface of eukaryotic cells, binding and blocking adhesive sites on the bacterial fimbriae. Other possible explanation is that mannose covers hydrophobic groups on the fimbriae, making the fimbriae more hydrophilic and thus repellent to other cells. On the other hand, fimbriae are allosteric proteins and binding of mannose induces a change from a hydrophobic and adhesive to a hydrophilic and non-adhesive form [53]. Although mannose has shown potential to prevent the attachment of *E. coli* strains to a very wide range of biotic surfaces (animal, plant and fungal cells) [54], the present study revealed that different results can be obtained for abiotic surfaces.

It was found that surface conditioning with myristic and palmitic acid can inhibit biofilm formation, but a dosage dependent effect was not observed. Although this may suggest that a saturation level has been reached, it has also been shown that changing the concentration of conditioning agent may not affect the extent of cell attachment [55]. Contact angle analysis showed that polystyrene hydrophobicity was slightly reduced upon conditioning with myristic and palmitic acid. Additionally, since the assay was conducted at a pH of 5, which is above the pKa values for both acids, these compounds will be anionic and therefore a cell repulsion effect may occur. These effects may have contributed to a lower cell adhesion. Whitehead et al. [56] also demonstrated in a previous work that the addition of organic material resulted in changes to the substratum physicochemistries at the surface/bacterial interface. To the best of our knowledge, there are no published studies about the individual effect of membrane fatty acids (such as myristic and palmitic acid) on *E. coli* biofilm formation. However, it has been demonstrated that exogenously added fatty acids modulate various bacterial activities, including motility, virulence, cell growth, and differentiation [57]. Soni et al. [58] showed that a fatty acid mixture (containing palmitic acid) negatively influenced *E. coli* K-12 biofilm formation since the long-chain fatty acids had ability to inhibit AI-2 based cell signaling. Regarding palmitic acid, its antimicrobial activity against oral bacteria at a similar concentration to that used in this work (0.025 g l^−1^) has already been reported [59]. Palmitic acid is not only capable of inhibiting *Pseudomonas aeruginosa* biofilm formation [60], but also of disrupting biofilms of *Candida albicans*, *P. aeruginosa* and *Bacillus pumilus* from glass surfaces [61]. Thus, it is likely that in this work some antibiofilm activity from the palmitic acid may have reduced *E. coli* adhesion and subsequent biofilm growth. Information about the action of myristic acid is even rarer and contradicts the results obtained in this work. This fatty acid repressed the swarming motility of *Proteus mirabilis*, however it slightly stimulated biofilm formation and EPS production in microtiter plates made of polyvinyl chloride [62].

Our research group has recently shown that conditioning the surface with cellular compartments (total cell extract, cytoplasm with cellular debris and periplasmic extract) can prevent *E. coli* biofilm formation under dynamic flow conditions [4]. The results obtained in this work with specific cell wall constituents reinforce that in food processing equipment where biofilm formation is not critical below a certain threshold, bacterial lysis and following adsorption of cell components to surface materials may reduce biofilm buildup and extend the operational time by increasing cleaning intervals.

## 5. Conclusion

It was demonstrated that *E. coli* cells are reservoirs of molecular compounds that may be very useful as effective inhibitors of bacterial adhesion. Furthermore, it is possible that the formation of problematic biofilms in industrial environments could be reduced through surface treatment with innocuous compounds such as myristic and palmitic acid. A better understanding of the mechanisms of bacterial adhesion and biofilm formation on abiotic surfaces will undoubtedly be gained from investigations leading to the isolation and chemical characterization of cellular compounds. This work also suggests that 96-well microtiter plates can simulate biofilm formation in flow systems as long as the shear stress is maintained, showing that flow topology at low shear stress values is comparatively less important.
